# The *Cstf2t* Polyadenylation Gene Plays a Sex-Specific Role in Learning Behaviors in Mice

**DOI:** 10.1371/journal.pone.0165976

**Published:** 2016-11-03

**Authors:** Jaryse C. Harris, Joseph M. Martinez, Petar N. Grozdanov, Susan E. Bergeson, Paula Grammas, Clinton C. MacDonald

**Affiliations:** 1 Department of Cell Biology & Biochemistry, Texas Tech University Health Sciences Center, Lubbock, Texas, United States of America; 2 Department of Pharmacology and Neuroscience, Texas Tech University Health Sciences Center, Lubbock, Texas, United States of America; 3 Department of Biomedical and Pharmaceutical Sciences, University of Rhode Island, Kingston, Rhode Island, United States of America; University of Nevada School of Medicine, UNITED STATES

## Abstract

Polyadenylation is an essential mechanism for the processing of mRNA 3′ ends. CstF-64 (the 64,000 *M*_*r*_ subunit of the cleavage stimulation factor; gene symbol *Cstf2*) is an RNA-binding protein that regulates mRNA polyadenylation site usage. We discovered a paralogous form of CstF-64 called τCstF-64 *(Cstf2t)*. The *Cstf2t* gene is conserved in all eutherian mammals including mice and humans, but the τCstF-64 protein is expressed only in a subset of mammalian tissues, mostly testis and brain. Male mice that lack *Cstf2t* (*Cstf2t*^*-/-*^ mice) experience disruption of spermatogenesis and are infertile, although female fertility is unaffected. However, a role for τCstF-64 in the brain has not yet been determined. Given the importance of RNA polyadenylation and splicing in neuronal gene expression, we chose to test the hypothesis that τCstF-64 is important for brain function. Male and female 185-day old wild type and *Cstf2t*^*-/-*^ mice were examined for motor function, general activity, learning, and memory using rotarod, open field activity, 8-arm radial arm maze, and Morris water maze tasks. Male wild type and *Cstf2t*^*-/-*^ mice did not show differences in learning and memory. However, female *Cstf2t*^*-/-*^ mice showed significantly better retention of learned maze tasks than did female wild type mice. These results suggest that τCstf-64 is important in memory function in female mice. Interestingly, male *Cstf2t*^*-/-*^ mice displayed less thigmotactic behavior than did wild type mice, suggesting that *Cstf2t* may play a role in anxiety in males. Taken together, our studies highlight the importance of mRNA processing in cognition and behavior as well as their established functions in reproduction.

## Introduction

Both reproductive and cognitive functions require extensive alternative mRNA processing to afford diverse gene expression in support of their unique physiologies. For example, alternative mRNA polyadenylation controls gene expression in many tissues [1, 2], particularly in testis [3] and brain [4, 5]. In testis, alternative polyadenylation alters expression of critical genes controlling spermatogenesis [6–10], while in brain it controls specific neuronal functions [11–16]. At least 80 proteins are known to be involved in mRNA polyadenylation in mammals [17]. Two important components, the cleavage and polyadenylation specificity factor (CPSF) and the cleavage stimulation factor (CstF), cooperate to recruit the rest of the polyadenylation machinery to the polyadenylation site [18]. While CPSF mediates both endonucleolytic cleavage [19] and recognition of the upstream polyadenylation signal [20–22], CstF appears to play a more regulatory role by choosing polyadenylation sites [23–25]. Additionally, components of both CPSF and CstF are known to be involved in regulation of cytoplasmic mRNA translation in synapses [26–28].

CstF is composed of three subunits, CstF-50, CstF-77, and CstF-64. CstF-64 (gene symbol *Cstf2*) is the 64,000 *M*_*r*_ RNA-binding subunit of CstF [29, 30] that recognizes the GU-rich element downstream of the site of cleavage [23, 31]. As such, CstF-64 is necessary for efficient polyadenylation of many genes [32]. In specific instances, it has been shown that changes in levels of CstF-64 are involved in control of alternative polyadenylation in immune cells [33, 34] and in other systems [35]. A neuronal-specific splice variant, βCstF-64, is expressed exclusively in vertebrate brains [36, 37], suggesting a conserved cognitive function for CstF-64.

CstF-64 has a paralog in mammals, τCstF-64 (gene symbol *Cstf2t*, [38, 39]), that is expressed most prominently in testis [40], but also in brain and other tissues [25, 41–43]. Male mice lacking *Cstf2t* are infertile, indicating that τCstF-64 is necessary for spermatogenesis through control of mRNA polyadenylation and suppression of expression of intergenic and non-coding genomic regions [44–47], and for alternative splicing of specific gene products in male germ cells [48]. Work from many laboratories has documented specific effects of altered mRNA polyadenylation on gene expression in brain, resulting in altered neuronal functions and behavior [4, 5, 13–16]. Because it is also expressed in brain, here we tested whether loss of *Cstf2t* had effects on behavior in mice. Casual observation of *Cstf2t*^*-/-*^ mice in their cages did not reveal overt changes in behavior [45]. However, we show here that systematic behavioral testing revealed significant differences in behavior between wild type and *Cstf2t*^*-/-*^ mice, specifically in memory-related behaviors. The *Cstf2t* gene did not have a significant effect on motor function in female mice, but *Cstf2t*^*-/-*^ males were more active than wild type males. More importantly, we found that female *Cstf2t*^*-/-*^ mice showed significantly better retention of learned spatial memory tasks compared to age-matched wild type female mice; males did not show this improvement. We also saw decreased thigmotactic swimming in *Cstf2t*^*-/-*^ male mice, suggestive of decreased anxiety in the absence of τCstF-64. These results suggest that the τCstF-64 protein encoded by *Cstf2t* may act to suppress spatial learning and memory in a sex-dependent manner through regulation of neural mRNA processing.

## Materials and Methods

### Animal Use and Generation of *Cstf2t*^*tm1Ccma*^ Mice

All animal treatments and tissues obtained in the study were performed according to protocols approved by the Institutional Animal Care and Use Committee at the Texas Tech University Health Sciences Center in accordance with the National Institutes of Health animal welfare guidelines. The named Institutional Animal Care and Use Committee (IACUC) specifically approved this study. TTUHSC’s vivarium is AAALAC-certified and has a 12-hour light/dark cycle (lights on at 0600 h) with temperature and relative humidity of 20–22°C and 40–70%, respectively.

Deletion of the entire *Cstf2t* coding region from chromosome 19, breeding, and genotyping were described previously [44]. Briefly, the knockout targeting vector was created using the *Cstf2t* coding region from chromosome 19 with pGK-Neo, electroporated into 129SvEv ES cells, and G418-resistant colonies in which Neo had replaced *Cstf2t* were identified by PCR. These cells were microinjected into C57BL/6 embryos and reimplanted into pseudopregnant females. Germ-line transmission was confirmed by PCR analysis of F1 animals. *Cstf2t*^*tm1Ccma*^ mice used in these studies were therefore of mixed C57BL/6-129SvEv background. Mutants were maintained as a congenic strain by repeated backcrossing (every 4–5 generations) to C57BL/6NCrl (Charles River) and otherwise breeding exclusively within the colony. At the time of this study, mice were bred to approximately 50 generations, with at least ten backcrosses to C57BL/6NCrl. Mice were housed individually in clear cages with wood chip bedding and, except where noted, had free access to food and water. For the duration of this study, females were housed apart from males to minimize hormone effects on behavior.

### Genotyping of *Cstf2t*^*tm1Ccma*^ Mice by PCR

Genomic DNA was extracted from tail snips of *Cstf2t*^*tm1Ccma*^ mice by proteinase K digestion followed by isopropanol precipitation. PCRs were performed using *Cstf2t*- and *Cstf2t*^*tm1Ccma*^-specific primers to determine the presence of the transgene [44].

### Protein isolation and immunoblots

Whole brain tissue from wild type or *Cstf2t*^-/-^ male and female adult mice (>12 months) was isolated and sonicated in radioimmunoprecipitation (RIPA) buffer [39, 43]. Protein concentration was measured using the BCA Protein Assay kit (Thermo). For testis, seminiferous tubules were isolated as follows: a small incision was made in the tunica albuginea of the testis and the contents (mainly seminiferous tubules) were gently collected in 5 mL of ice-cold Dulbecco’s Phosphate-Buffered Saline (DPBS, Life Technologies) supplemented with phenylmethanesulfonyl fluoride (PMSF). The contents were vigorously shaken, breaking apart the tissue, allowed to settle at unit gravity for 5 min on ice, and the supernatant (containing mainly Leydig cells) was aspirated. The procedure was repeated 3 times. After the final settlement, the tissue was centrifuged briefly at 400×g. Seminiferous tubules from one animal were lysed in 250 μl of extraction buffer (DPBS, 0.5% Triton X-100 (v/v), 2 mM PMSF, 0.02% NaN_3_), sonicated briefly, incubated on ice for 10 min, and centrifuged for 10 min at 400×g at 4°C. Equal amounts of protein from wild type and *Cstf2t*^*-/-*^ animals were loaded on pre-cast NuPAGE Novex 4–12% Bis-Tris Gels (Life Technologies), followed by a semi-dry transfer onto polyvinylidene fluoride membranes. Membranes were incubated with primary antibody as indicated, followed by the appropriate secondary antibody conjugated to horseradish peroxidase. Mouse anti-CstF-64 (3A7), either a mouse anti-τCstF-64 monoclonal (6A9) or polyclonal (Bethyl A301-487A) antibody were used as described [40, 41]. The E7 anti-β-tubulin monoclonal antibody obtained from the Developmental Studies Hybridoma Bank was used at a dilution of 1:1000.

### Rodent neurons and glial cells

Primary neurons were isolated from 18-day old rat pups of mixed sexes [49]. The glia cell extract was from the C6 cell line derived from rats [50].

### Numbers of animals used for behavioral analyses

For behavioral experiments, male and female *Cstf2t*^*+/+*^ (wild type) and *Cstf2t*^*-/-*^ mice aged 185 ± 10 days were used. Genotyping was performed using tail snips and PCR as previously described [44]. The numbers for each group are as follows:

Mice aged 185 ± 10 days (M/F P185):

- Male *Cstf2t*^-/-^ mice, n = 16
- Male wild type, n = 15
- Female *Cstf2t*^-/-^ mice, n = 17
- Female wild type mice, n = 20

### Activity and Locomotor Assessments

The Open Field Activity Assay used to assess general motility consisted of a 43 × 43 × 43 cm open top box with clear Plexiglas sides (Med Associates, Inc., St. Albans, Vermont). Infrared (IR) sensors tracked the animals’ movement within the chamber using Med Associates Activity Monitor Software Version 5. Opaque black paper was affixed to the outside of the chamber to ensure each animal had a similar visual field. The day prior to testing, the animals were allowed to explore the assay box in order to acclimate. To start testing, the animal was placed in the center of the box facing away from the technician and allowed to explore freely for 5 minutes while being tracked by the IR sensors. Before and after each trial, the box was cleaned with 70% ethanol to help ensure a consistent scent environment. The mazes were thoroughly cleaned with Quatra-Cide at the conclusion of maze testing each day.

To assess general motor function, a single mouse rotarod treadmill (Med Associates, Inc.) was used. The animal was placed on the rotarod facing away from the technician and the apparatus set to gradually increase rotation from 4 rpm to 40 rpm over 300 seconds [51]. When the animal falls, an IR beam is broken, stopping the motor and the timer. The time for each trial was recorded, with a maximum of 300 seconds. Each animal performed three trials a day for 3 consecutive days. If the animal reached 300 seconds, that animal did not participate in any of the remaining trials for that day.

### 8-arm Radial Arm Maze

Prior to radial arm maze training, mice were placed on restricted diets so that each mouse maintained a weight no less than 85% of its free-feeding weight at the start of experiments to motivate foraging for food in the maze [52]. A modular Plexiglas radial arm maze (RAM) with a central compartment of 20.3 cm and eight arms of 35.5 cm was from Med Associates, Inc. Food receptacles were located at the end of each arm. Visual cues were placed in a cross pattern outside the RAM. The technician always remained in the same location, serving as a visual cue. Entry into an arm was recorded when all four paws crossed from the center compartment into an arm; an entry was coded as correct when the arm had not been previously entered during the current session. After each trial, the maze was cleaned with 70% ethanol. The total number of arm entries required for completion of the maze was used to evaluate overall accuracy of learning and memory; the number of consecutive correct entries before first error was used to estimate spatial memory span, an estimate of working memory. The maze was cleaned thoroughly with Quatra-Cide at the end of maze testing each day.

Initially, three 10-minute sessions were conducted during which the mice were placed in the center of the maze and allowed to explore freely. The maze was baited with a drop of sweetened condensed milk at the end of each arm to encourage exploration. To start each trial, the animal was placed in the center of the baited maze facing away from the technician. Mice were then allowed 5 minutes to retrieve the bait from each of the eight arms. A session ended when the mouse had visited each of the eight arms at least once or the maximum time limit of 5 minutes had elapsed. Mice that did not complete the maze task in the allotted time were marked as incomplete. Mice were tested daily at approximately the same time for four 5-day blocks with two days of rest between blocks.

### Morris water maze

The water maze apparatus (Coulborn Instruments, Allentown, PA) based on the design of Morris [53] was used to assess spatial and reference memory (retention of the learned maze task) in wild type and *Cstf2t*^-/-^ mice. The Morris water maze (MWM) consisted of a 183 cm diameter pool filled with water to a depth of 48 cm. Water was maintained at 20°C. An 18 cm diameter platform was placed in the pool approximately 2 cm below the surface of the water in one of the quadrants. Four unique visual cues were placed around the inside wall of the pool in a cross pattern. The water was made opaque to obscure the platform location using non-toxic white tempera paint (Utrecht Art Supplies, Cranbury, NJ). Tracking software (Actimetrics, Wilmette, IL) was used to monitor and record each trial. The MWM paradigm consisted of four 60-second trials per day for 5 consecutive days followed by a reference memory probe trial at 24 hours after training. The inter-trial interval for each mouse was approximately 10 minutes. For each trial, the animal was placed in the pool at one of four pre-determined start positions and allowed to swim while being tracked by the software. The trial ended when the animal found the platform or when 60 seconds had elapsed. If the mouse did not reach the platform, it was placed onto the platform and left for 10 seconds. After each trial, the animal was dried, transferred to a warming box lined with absorptive material, and left until the completion of the next animal’s trial. Reference memory probe trials consisted of a single 60-second free swim from a single start point. The platform was removed for the probe trial, but its previous location was recorded with the monitoring software.

### Statistical Analyses

For behavioral experiments, 1- or 2-way ANOVA analyses followed by post hoc Tukey’s multiple comparison tests to compare different groups, linear regression analysis, or student’s t-test were performed where appropriate. Graphical presentation and statistical analysis were performed using the software Prism version 6.0 (GraphPad Inc., San Diego CA). Significance was determined at p<0.05.

## Results

### The τCstF-64 polyadenylation protein is expressed in both neurons and glia in wild type brain in both male and female mice

**Fig 1:**
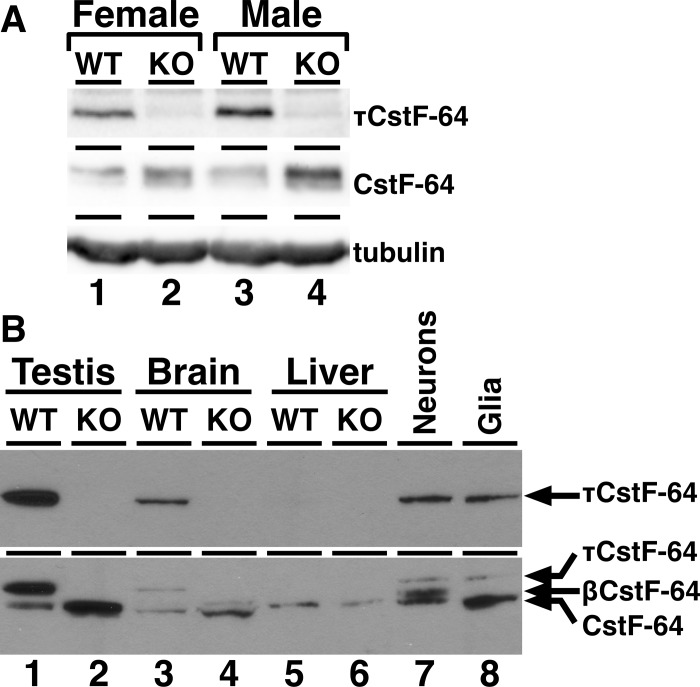
τCstF-64 and CstF-64 are expressed in brains of adult mice. (A) Brain tissue extracts were separated by 10% SDS-PAGE and probed with antibodies that recognized τCstF-64 (Bethyl A301-487A, top panel), CstF-64 (3A7, middle panel), or β-tubulin (E7, bottom panel). Lanes were loaded with 10 μg total brain extract; lanes 1, female wild type; lanes 2, female *Cstf2t*^*-/-*^; lanes 3, male wild type; lanes 4, male *Cstf2t*^*-/-*^. (B) Testis (lanes 1, 2), brain (lanes 3, 4), and liver (lanes 5, 6) from wild type (lanes 1, 3, 5) or *Cstf2t*^*-/-*^ (lanes 2, 4, 6) male mice were separated by SDS-PAGE as in 1A. Extracts from primary rat neurons (lane 7) and rat C6 glioma cells (lane 8) were included for comparison. Blots were probed with anti-τCstF-64 (6A9, top panel), and then subsequently re-probed with anti-CstF-64 (3A7, bottom panel) antibodies. Apparent migrations of τCstF-64, CstF-64, and βCstF-64 are indicated by arrows.

To confirm expression of τCstF-64 in brains of wild type, but not *Cstf2t*^*-/-*^, male and female mice, total brain tissue was isolated from adult mice, separated on SDS-PAGE, and probed with antibodies to τCstF-64 and CstF-64 (Fig 1A). As previously reported [40, 41, 44], τCstF-64 was detected in brain tissue of male wild type mice (top panel, lane 3) but was not detected in *Cstf2t*^*-/-*^ males (lane 4). Likewise, τCstF-64 expression was detected in brain tissue of female wild type (lane 1) but not *Cstf2t*^*-/-*^ (lane 2) mice. This demonstrates that τCstF-64 is expressed at similar levels in both male and female mice. CstF-64 was detected at in brain tissue of all mice examined (middle panel). The apparent increase in CstF-64 in *Cstf2t*^*-/-*^ samples (lanes 2, 4) is a documented post-transcriptional phenomenon whereby loss of τCstF-64 results in increased CstF-64 [43, 44, 48, 54]. β-Tubulin was used to control for sample loading (bottom panel).

Next, we examined the distribution of τCstF-64 in extracts from testis, brain, and liver from wild type and *Cstf2t*^*-/-*^ [44] male mice (Fig 1B, lanes 1–6). τCstF-64 was detected in extracts from wild type mouse testis and brain (lanes 1 and 3), but not in liver (lane 5), consistent with earlier reports using the 6A9 antibody [40–42]. τCstF-64 was detected at similar levels in both neurons isolated from wild type juvenile rats (lane 7) and in C6 rat glial cells [50], suggesting that τCstF-64 is distributed equally between neurons and glia in rodent brain. *In situ* hybridization detection from the Allen Brain Atlas shows strong expression in the hippocampus and dentate gyrus of C57BL/6J mouse brain (S1 Fig).

Subsequently, we re-probed the same immunoblot with the 3A7 antibody that detects CstF-64 but not τCstF-64 in mice [38–41, 44]; thus both τCstF-64 and CstF-64 isoforms were visualized in this blot (Fig 1B). As expected, a band corresponding to the apparent migration of CstF-64 was observed in every tissue examined (Fig 1B, lanes 1–6). Similarly, CstF-64 was expressed in isolated wild type rat neuronal and glial cells (lanes 7, 8). This confirmed expression of both CstF-64 and τCstF-64 in testis and brain [40–42]. The apparent slower migration of CstF-64 in the samples from liver (lanes 5, 6) has been observed previously [36, 40, 41, 45]. We speculate that these might be due to post-translational modifications (e. g., phosphorylation), though this has not been confirmed. Interestingly, we saw a third 3A7-reactive band in neurons (lane 7) that was also faintly visible in wild type and *Cstf2t*^*-/-*^ brain (lanes 3, 4). Most likely, this is the brain-expressed βCstF-64 splice variant of CstF-64 reported earlier [36, 37]. As βCstF-64 was not visible in glia, we confirm that it is neuron-specific.

### *Cstf2t*^*-/-*^ mice respond normally to visual cues

To ensure that the mice could find the platform in the Morris water maze and to eliminate the possibility that visual impairment was confounding our tests, we completed a simple test of general visual acuity. Mice were placed in the maze facing away from the platform, which was marked with a flag. On every occasion, regardless of genotype, an optokinetic reflex was apparent with recognition of the flag and immediate swimming to the platform took place. We concluded that the mice could adequately see the cues in the maze.

### *Cstf2t*^*-/-*^ and wild type female mice show no differences in general activity or gross locomotor function

**Fig 2:**
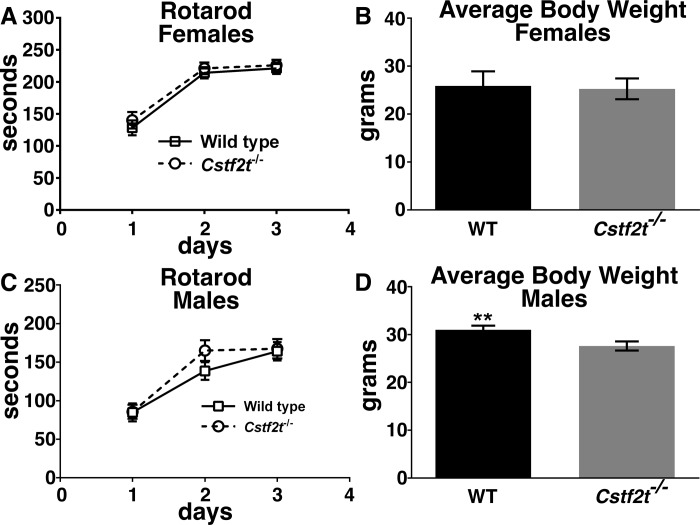
Gross locomotor performances of *Cstf2t*^-/-^ and wild type mice are not significantly different. Female and male *Cstf2t*^-/-^ and wild type mice (185 ± 10 days of age) were run on a single mouse rotarod 3 times per day for 3 days to assess gross locomotor function. **(A and C)** Comparison of female and male mouse groups’ performance on rotarod indicated significant differences between groups (2-way ANOVA (F (3,528) = 27.78; p<0.0001). A post-hoc Tukey’s multiple comparison test indicated no significant differences between female wild type and *Cstf2t*^*-/-*^ and male wild type and *Cstf2t*^*-/-*^ mice. **(B and D)** Comparison of female and male mice indicated a group difference (1-way ANOVA (F (7, 123) = 23.36; p<0.0001. **(B)** A post-hoc Tukey’s multiple comparison of female groups indicated no significant differences between female *Cstf2t*^-/-^ and female wild type mouse weights **(D)** but did indicate a significant difference between male wild type and male *Cstf2t*^-/-^ mouse weights (** p<0.01). All data are presented as mean ± SEM. ANOVA analyses were conducted using all groups.

All female mice demonstrated similar performances on the rotarod (Fig 2A) and showed no significant differences in general activity (S2 Fig). No differences in weight between *Cstf2t*^*-/-*^ and wild type female mice were observed (Fig 2B). These data suggest that any differences observed in performance of subsequent behavioral tasks by female mice were not due to differences in locomotor ability or general animal activity.

### *Cstf2t*^*-/-*^ males are more active than wild type males, but do not show differential locomotor function

Using a rotarod to test gross locomotor function [51], wild type and *Cstf2t*^-/-^ males performed similarly during three days of testing (Fig 2C), indicating similar gross locomotor function between age matched genotypes. However, when a 43 × 43 × 43 cm chamber was used to assess general open field activity of the mice. Male *Cstf2t*^*-/-*^ mice retained higher activity levels in the six measures while the wild type mice lost activity (S2 Fig). This suggests that in males, *Cstf2t* may contribute to a loss of activity, and in its absence, male mice retain higher levels of activity. Male *Cstf2t*^*-/-*^ mice were lighter than wild type males of the same age (Fig 2D), which might have contributed to their greater overall activity. Future mechanistic experiments may clarify this difference. The weight difference between wild type and *Cstf2t*^*-/-*^ male mice did not appear to affect rotarod performance.

### Female and male mice demonstrated similar spatial learning and memory spans in the 8-arm radial arm maze task

**Fig 3:**
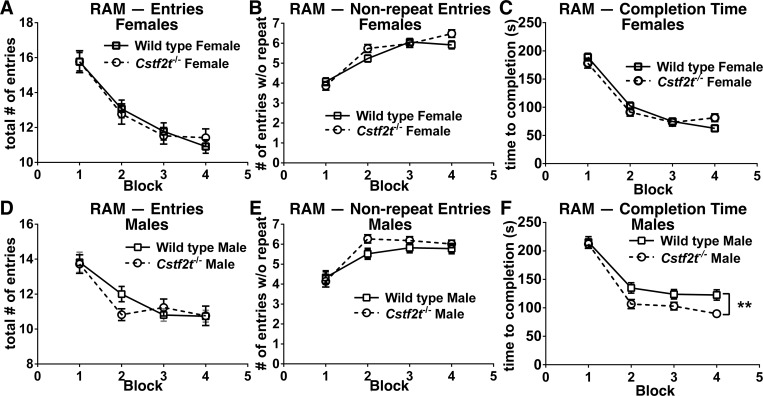
Female wild type and *Cstf2t*^*-/-*^ mice display similar working and spatial memory as assessed by 8-arm radial arm maze (RAM). Male and female *Cstf2t*^-/-^ and wild type mice were run in a RAM once per day in five-day blocks with two days break between blocks for four weeks. **(A and D)** Comparison of **(A)** female and **(D)** male performance using “total # of entries,” an indicator of overall accuracy of learning and memory showed no significant differences between groups (2-way ANOVA (F (3, 1146) = 6.836; p<0.001)). A post-hoc Tukey’s multiple comparison test confirmed no differences between female and male *Cstf2t*^-/-^ and wild type groups, but indicated significant differences between female and male groups. **(B and E)** Comparison of **(B)** females and **(E)** males using the “total # of entries without error” metric, an estimate of memory span and, in turn, an estimate of working memory showed no significant differences between groups (2-way ANOVA (F (3, 1154) = 1.901; p = 0.13)). **(C and F)** Comparison of all groups examining “time to completion” of the maze task showed significant differences between groups in the “time to completion” metric (2-way ANOVA (F (3, 1151) = 27.99; p<0.0001)). A post-hoc Tukey’s multiple comparison test revealed no significant differences between **(C)** females, but revealed significant differences in **(F)** male wild type and *Cstf2t*^-/-^ mice (**p<0.001). All data are presented as mean ± SEM. ANOVA analyses were conducted using all groups.

In the 8-arm radial arm maze, each arm is baited with a small amount of food, and the mouse is placed in the center and allowed to roam the maze until a set number of arm choices has been made or a fixed amount of time has passed [52]. After training, both wild type and *Cstf2t*^*-/- *^mice of both sexes required the same total number of arm entries to complete the 8-arm radial arm maze task, although males required fewer entries in the first block (Fig 3A and 3D). This demonstrated similar spatial learning and memory for all mice. All groups showed no significant differences in total number of correct arm entries before an error (Fig 3B and 3E), and had similar numbers of incompletions across the period of testing (S3 Fig), though wild type males took significantly longer to complete the radial arm maze task than the other mice groups (Fig 3F). This might reflect the decreased overall activity of the wild type male mice (S2 Fig).

### Female *Cstf2t*^*-/-*^ mice exhibit better spatial learning in the Morris water maze navigation task than their wild type counterparts

**Fig 4:**
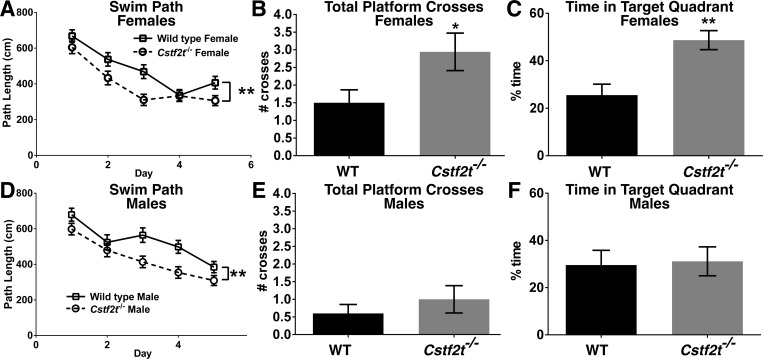
Both male and female mice show differences in average swim distances between wild type and *Cstf2t*^*-/-*^ mice, while female *Cstf2t*^*-/-*^ mice display significantly better reference memory 24 hours after Morris water maze training. Female and male *Cstf2t*^-/-^ and wild type mice were trained to find a submerged platform in a Morris water maze (MWM) four times per day for 5 days followed by reference memory trials 24 hours post training, during which the platform was removed. **(A and D)** Distance swam (in cm) to reach the escape platform between wild type and *Cstf2t*^*-/-*^ **(A)** female and **(D)** male mice was examined using a 2-way ANOVA (F (3, 1340) = 13.16; p = 0.0001), indicating significant group differences. A post-hoc Tukey’s multiple comparison test indicated significant differences between both female and male wild type and *Cstf2t*^*-/-*^ mice (**p<0.001). **(B and E)** Total number of platform crosses (how many times a mouse swam over the escape platform’s previous location) 24 hours after the last training day. **(B)** Female *Cstf2t*^*-/-*^ mice showed a significantly greater number of platform crosses than female wild type mice using a two-tailed Student’s t-test (p<0.05). **(E)** There was no significant difference in total number of platform crosses between wild type and *Cstf2t*^*-/-*^ male mice (Student’s t-test). **(C and F)** Percent of time spent in the target quadrant (how much of the reference trial period the mouse spent in the maze quadrant where the escape platform used to reside). **(C)** Female *Cstf2t*^*-/-*^ mice showed a significantly greater amount of time in the target quadrant than female wild type mice (two-tailed Student’s t-test, p<0.001). **(F)** There was no significant difference in total number of platform crosses between male wild type and male *Cstf2t*^*-/-*^ mice (Student’s t-test)). All data are presented as mean ± SEM. *p<0.05, **p<0.001. ANOVA analyses were conducted using all groups.

In the water navigation task, which is primarily a test of spatial learning and spatial memory [53, 55], mice were challenged to find a hidden subsurface platform in one quadrant of a 183 cm diameter pool based on visual cues in each quadrant. As assessed by total distance swam to reach the escape platform, all groups learned the water maze task between day 1 and day 5 of training (Fig 4A and 4D, and S3 Fig). However, both male and female *Cstf2t*^*-/-*^ mice demonstrated reduced path lengths (Fig 4A and 4D) after five days of training compared to their wild type counterparts, suggesting that the *Cstf2t*^*-/-*^ mice learned this spatial task better than the wild type mice. Both female and male *Cstf2t*^*-/-*^ mice had slower swim speeds than their wild type counterparts (S4 Fig). These effects do not correlate with altered locomotor functions (Fig 2A and 2C, and S2 Fig), so they might reflect an improved adaptation strategy for the *Cstf2t*^*-/-*^ mice overall [56].

### Female *Cstf2t*^*-/-*^ mice showed significantly better retention of the learned water navigation task than wild type mice

To test recollection of learned spatial tasks, we trained mice to locate the escape platform in the water navigation task for five days, then removed the platform and tested the mice for recall of the previous location at 24 hours after the completion of day 5 maze training. At 24 hours, female *Cstf2t*^*-/-*^ mice demonstrated significantly better retention of the platform location as assessed by the “Total Platform Crosses” (Fig 4B) and “Time in Target Quadrant” (Fig 4C) metrics compared to their wild type counterparts. In contrast, male *Cstf2t*^*-/-*^ mice demonstrated no better retention of the maze task than male wild type mice in the two metrics examined (Fig 4E and 4F). This suggests that *Cstf2t* effects female, but not male mice’s abilities in recollection of learned spatial tasks.

### *Cstf2t*^*-/-*^ male mice exhibited less thigmotaxis (an indicator of anxiety) than wild type controls in the Morris water maze navigation task

**Fig 5:**
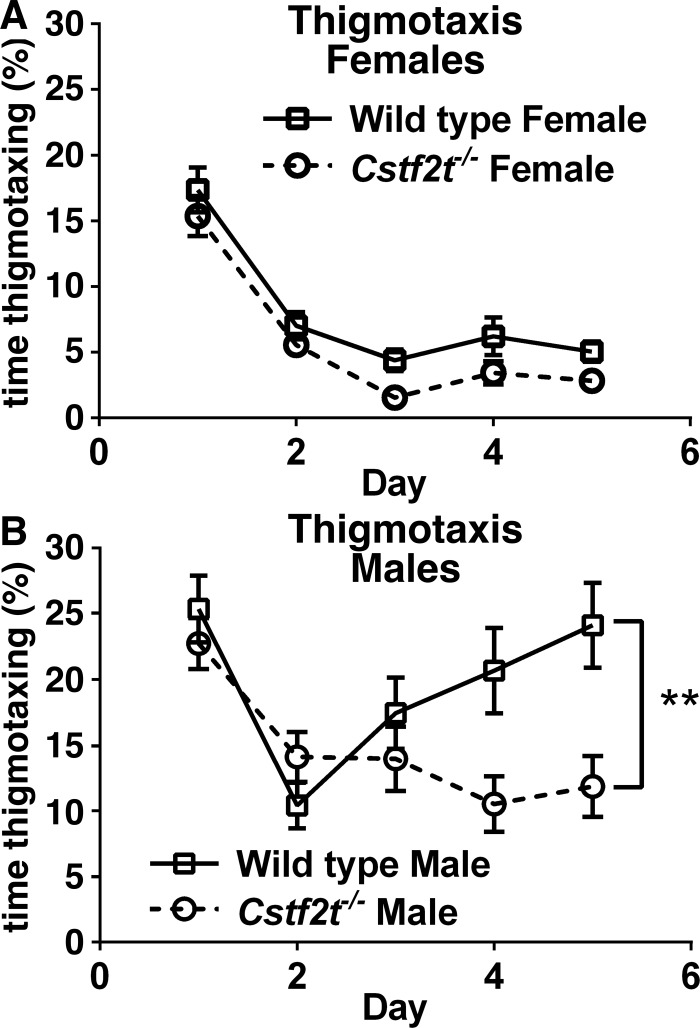
Male and female *Cstf2t*^*-/-*^ mice show reduced thigmotactic behavior, but males show it to a greater extent. **(A and B)** Amount of time spent in thigmotaxis (activity while remaining near the wall), expressed as a percentage of total time. **(A)** Female *Cstf2t*^*-/-*^ mice showed no difference in thigmotaxis compared to female wild type mice. **(B)** Male *Cstf2t*^*-/-*^ mice showed significant reduction of thigmotaxis compared to male wild type mice (2-way ANOVA (F (3, 1328) = 59.38; p<0.0001). A post-hoc Tukey’s multiple comparison test indicated significant differences between male *Cstf2t*^*-/-*^ mice and male wild type mice (p<0.001). All data presented as mean ± SEM. **p<0.001. ANOVA analyses were conducted using all groups.

The Morris water maze, in addition its ability to measure learning, can also expose other behavioral patterns indicative of movement control, cognitive mapping, and anxiety. Thigmotaxis, a behavioral taxis by which animals tend to stay close to the walls of a maze, is an indication of anxiety [57]. Female wild type and *Cstf2t*^-/-^ mice showed less thigmotaxis than their male counterparts by the end of the water navigation testing (Fig 5A), spending ~15% of total testing time in thigmotaxis on the first day of maze testing, which reduced to ~5% thigmotaxis by day 5.

Overall, male mice displayed more thigmotaxis than females in the water navigation task (Fig 5B). Both wild type and *Cstf2t*^-/-^ male mice maintained greater than 10% thigmotaxis for all five days of the water navigation task. However, thigmotaxis in male *Cstf2t*^-/-^ mice decreased to ~10% of total testing time by day 5, while wild type maintained significantly higher levels of thigmotaxis. This suggests that male *Cstf2t*^*-/-*^ mice were able to acclimate to the test better than their wild type brothers, and displayed less apparent anxiety while doing so.

## Discussion

τCstF-64 is an important protein associated with mRNA polyadenylation, which is a major controller of gene expression in specific tissues, particularly in testis and brain [3, 5, 10, 13, 58, 59]. The most common mechanism by which polyadenylation affects gene expression is by shortening or lengthening mRNA 3′ ends, thus hiding or revealing RNA control elements in the 3′ untranslated regions [5, 60], although it can also change protein isoforms [34, 61]. Targeted disruption of the testis-expressed polyadenylation gene *Cstf2t* resulted in severe male infertility, demonstrating a critical necessity for polyadenylation and mRNA processing in spermatogenesis [44, 45, 47]. The majority of the altered gene expression in spermatogenic cells in *Cstf2t*^*-/-*^ mice is likely due to altered polyadenylation, but altered splicing [48] and genome expression [46] also contribute. Because τCstF-64 is expressed at high levels in brain as well as testis [40–42], we wanted to determine whether *Cstf2t* influenced behavior as well as male fertility. We show here that τCstF-64 is expressed at similar levels in both male and female mice, and in both neurons and glia (Fig 1). This suggests the that any behavioral effects of τCstF-64 might be split between neuronal synaptic functions and glial plasticity functions [62]. It further supports a potential role for τCstF-64 in sexually dimorphic behavioral responses; for example, in the glial response to how different sexes display hormonal responses to injury [63]. We also confirm that the brain-specific splicing variant of CstF-64, βCstF-64 [36, 37] is exclusive to neurons (Fig 1B). These findings support the premise that CstF-64 and its paralogs, especially *Cstf2t* are potentially important for multiple brain functions through actions in both neurons and glia.

From previous casual observation of the mice in their cages, we saw no overt changes in behaviors in the *Cstf2t*^*-/-*^ mice [44, 45]. However, specific tests revealed alterations in spatial learning, spatial memory, and anxiety indicators had different penetration in males and females. Interestingly, in male germ cells, τCstF-64 regulates both alternative splicing and polyadenylation of *Crem*, one of the major targets of τCstF-64 in testis, resulting in differential expression of CREM protein isoforms that control downstream transcriptional targets [48]. Thus, we hypothesize that *Crem* might be one of the important targets that is affected in brain of *Cstf2t* in mice. Further, given the localization of *Cstf2* to the hippocampus and dentate gyrus, as seen in the Allen Brain Atlas [64] (S1 Fig), it is also easy to speculate about the effect of the gene in learning- and memory-related behaviors.

It seems incongruous that disruption of a gene for mRNA 3′ end processing such as *Cstf2t* would result in an increase in learning and memory while reducing anxiety. One function of τCstF-64 is to promote polyadenylation at proximal sites over distal sites [25, 65]. More specifically, a recent study demonstrated that CstF-64 cooperates with hnRNP H to regulate acetylcholinesterase isoforms in human neuronal cells [66], suggesting that τCstF-64 might play a similar role. Further, it has been shown that alternative 3′ exons participate in localization of mRNAs within neurons, for example to neural projections [67]. These suggest mechanisms by which *Cstf2t* could affect learning, memory, and anxiety through alternative polyadenylation and splicing [48], which would increase plasticity and learning. However, the current work suggests the opposite model: *Cstf2t* acts to suppress—though not eliminate—spatial learning and memory while enhancing anxiety. We speculate, therefore, that *Cstf2t* participates in a balance of increasing anxiety to reduce risk-taking activities, while reducing aspects of learning and memory that enable such activities. Perhaps there are specific mechanisms enabled by *Cstf2t* in the amygdala that enhance functional anxiety, but that suppress learning and memory in the hippocampus. Future experiments will test these and similar hypotheses.

Female and male rodents have demonstrated sex differences in molecular and behavioral memory formation as well as sex differences in the roles synaptic kinases and gene transcription play in memory formation. Additionally estrogen levels play a role in memory as do sexually dimorphic epigenetic mechanisms [68]. In our study, female *Cstf2t*^*-/-*^ mice demonstrated far better recollection of the maze task than wild type females (Fig 4), while there was no difference between the male mouse groups. This result is remarkable; although there is clearly a deleterious effect of the absence of *Cstf2t* in males (*Cstf2t*^*-/-*^ males are infertile and thus cannot pass on the trait [44]), there appear to be advantageous behavioral effects in females. Improved spatial learning and memory are survival traits in mice; for example, improved learning would allow mice to recognize and escape predators, while improved spatial memory would allow recollection of locations of food. This suggests to us that there might be evolutionary pressure to maintain a *Cstf2t*^*null*^ allele in a population such that improved learning and memory in homozygotic females would offset the deleterious effects of homozygotic male infertility. Earlier, we noted an overdominance of *Cstf2t*^*tm1Ccma*^ in testis (heterozygous males had greater numbers of germ cells than either of the homozygous groups [45]). This suggests the possibility that loss-of-function alleles in *Cstf2t* might display overdominance in spatial learning, as well, which could keep the allele in a population despite disadvantages to homozygotes.

*Cstf2t*^*-/-*^ males and females showed less thigmotaxis, which is often an indication of anxiety, than wild types (Fig 5B). *Cstf2t*^*-/-*^ males also required reduced times to finish the radial arm maze task (Fig 3F) and increased activity in the open field test (S2 Fig), behaviors that could be explained by decreased anxiety. These effects were not seen in females. Sex-related changes in anxiety are well-documented [69] and might depend at least in part on glutamate-receptor mediated pathways [70], serotonin uptake [71, 72], or defects in microglia [73]. Both male and female mice deficient in *Crem* display decreased anxiety [74], in a manner similar to *Cstf2t*-deficient mice (S2 Fig). *Cstf2t* controls *Crem* in testis at both the polyadenylation and splicing levels [48], which suggests that alteration in *Crem* expression might play a role in brain as well. We will test this hypothesis in future studies.

Furthermore, alterations in anxiety-indicating behaviors showed a strong sex bias. Given its role in control of global genome expression [46], it seems that *Cstf2t* could have profound epistatic effects on numerous functions, especially if the effects are on regulatory proteins with roles in chromatin expression. We have not yet uncovered a mechanism of how loss of the τCstF-64 polyadenylation protein might influence this type of behavior or why it might be most evident in males. Functionally, we speculate that males lacking *Cstf2t* might take greater risks in seeking social interaction or foraging for food, which could result in greater mortality due to predation. Why, then, would *Cstf2t* remain in the population? Ignoring, for the moment, that *Cstf2t*^*-/-*^ males are infertile [44], we can only speculate that pathways of anxiety have an unknown benefit in males, possibly in avoidance of dangerous situations.

Finally, we note that the behavioral differences found in *Cstf2t*^*-/-*^ mice (males and females) might have multiple causes due to the multiple levels at which τCstF-64 affects gene expression. While τCstF-64 is nominally a polyadenylation protein [25, 39, 54], it also appears to influence splicing [48] and global genome expression [46], making it an epigenetic modifier. We speculate that *Cstf2t* may be acting at all these levels in its effects on mouse behaviors. We plan to test this hypothesis in future work.

## Supporting Information

S1 Fig In situ hybridization data from the Allen Brain Atlas indicate that *Cstf2t* expression is strong and highest in the hippocampus and dentate gyrus. Sagittal section of C57BL/6J mouse brain tested using in situ hybridization (ISH) with a *Cstf2t* probe is shown on the left, with close-ups of the hippocampus and dentate gyrus shown on the right (A) Original ISH. (B) Pseudocolor ISH. The data can be independently identified at the Allen Brain Atlas (63). See: <http://mouse.brain-map.org/experiment/show/68196929>.
(TIF)

S2 Fig Gross locomotor performance of male and female *Cstf2t*^-/-^ and wild type mice in Open Field Activity. Values represent mean ± SEM. 2-way ANOVA; Tukey’s post-hoc tests were completed. *p<0.05, *Cstf2t*^*-/-*^ vs. wild type mice. Parameters were Distance Traveled, Time Ambulatory, Time Resting, Average Speed, Vertical Counts, and Jump Counts.
(TIF)

S3 Fig Female and male mice working and spatial memory as assessed by 8-arm radial arm maze (RAM). (A) Comparison of female groups using the “number of incomplete trials” metric, an estimate of maze task participation. There were no significant differences between groups. (B) Comparison of male groups examining “number of incomplete trials.” There were no significant differences between groups.
(TIF)

S4 Fig Spatial learning and memory as assessed using the Morris water maze (MWM). Female and male *Cstf2t*^-/-^ and wild type mice (185 ± 10 days of age) were trained in a MWM four times per day for 5 days followed by reference memory trials 24 hours post training. (A) Examination of differences in average group swim speed between female groups showed a significant difference between groups (2-way ANOVA (F (3, 1320) = 35.00; *p* < 0.0001)). A post-hoc Tukey’s multiple comparison test revealed a significant difference between *Cstf2t*^-/-^ and wild type mice (*p* < 0.0001). (B) Examination of differences in average group swim speed between male groups showed a significant difference between groups (2-way ANOVA (F (3, 1240) = 19.30; *p* < 0.0001)). A post-hoc Tukey’s multiple comparison test revealed significant differences between wild type mice and *Cstf2t*^-/-^ mice (*p* < 0.0001). All data are presented as mean ± SEM. Data shown are for 185 groups only. ANOVA analyses were performed on all groups.
(TIF)
