# Supplementary figures and images for: The Cstf2t Polyadenylation Gene Plays a Sex-Specific Role in Learning Behaviors in Mice
Source: PLoS One. 2016 Nov 3;11(11):e0165976. doi: 10.1371/journal.pone.0165976 (PMC5094787; doi:10.1371/journal.pone.0165976)

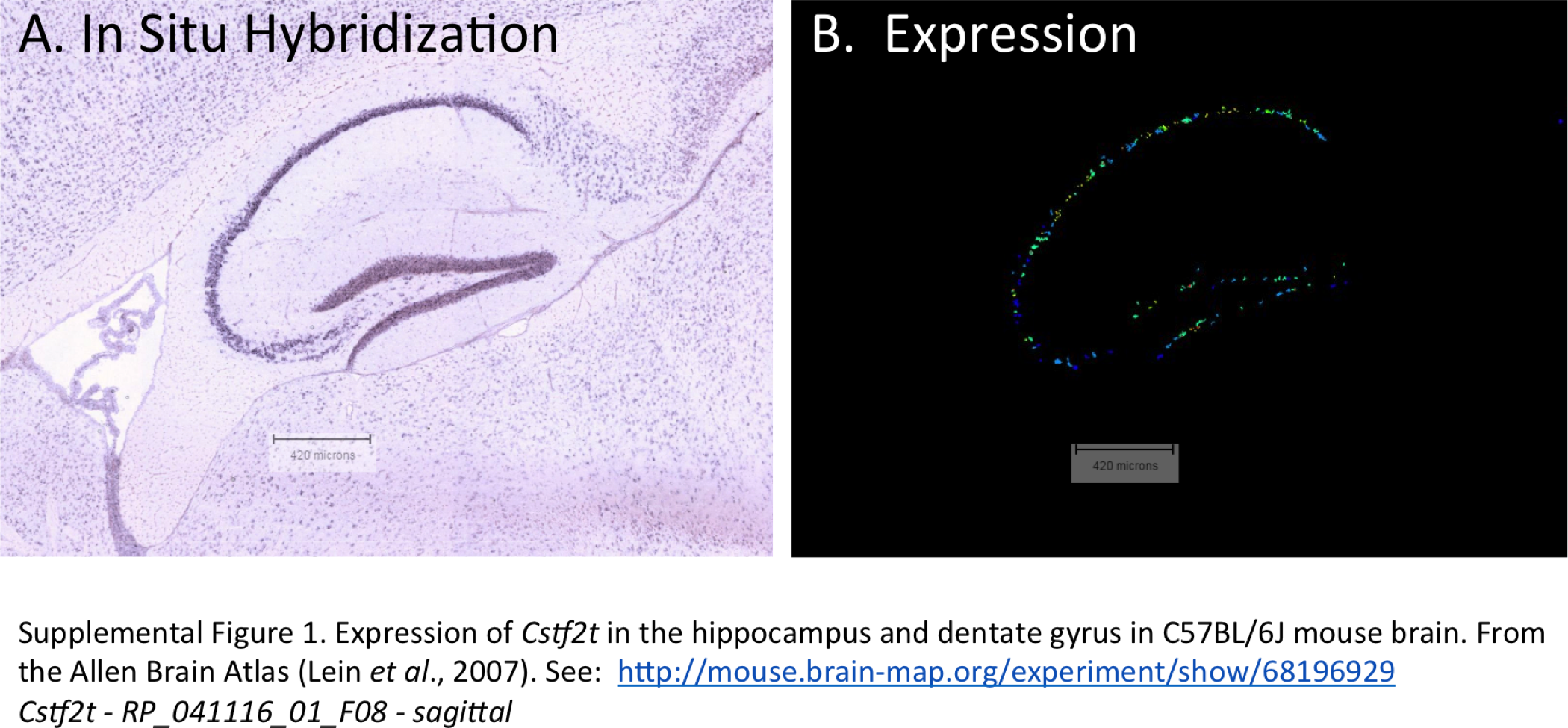

Supplement: S1 Fig — Sagittal section of C57BL/6J mouse brain tested using in situ hybridization (ISH) with a Cstf2t probe is shown on the left, with close-ups of the hippocampus and dentate gyrus shown on the right (A) Original ISH. (B) Pseudocolor ISH. The data can be independently identified at the Allen Brain Atlas (63). See: <http://mouse.brain-map.org/experiment/show/68196929>. (TIF) [file pone.0165976.s001.tif]

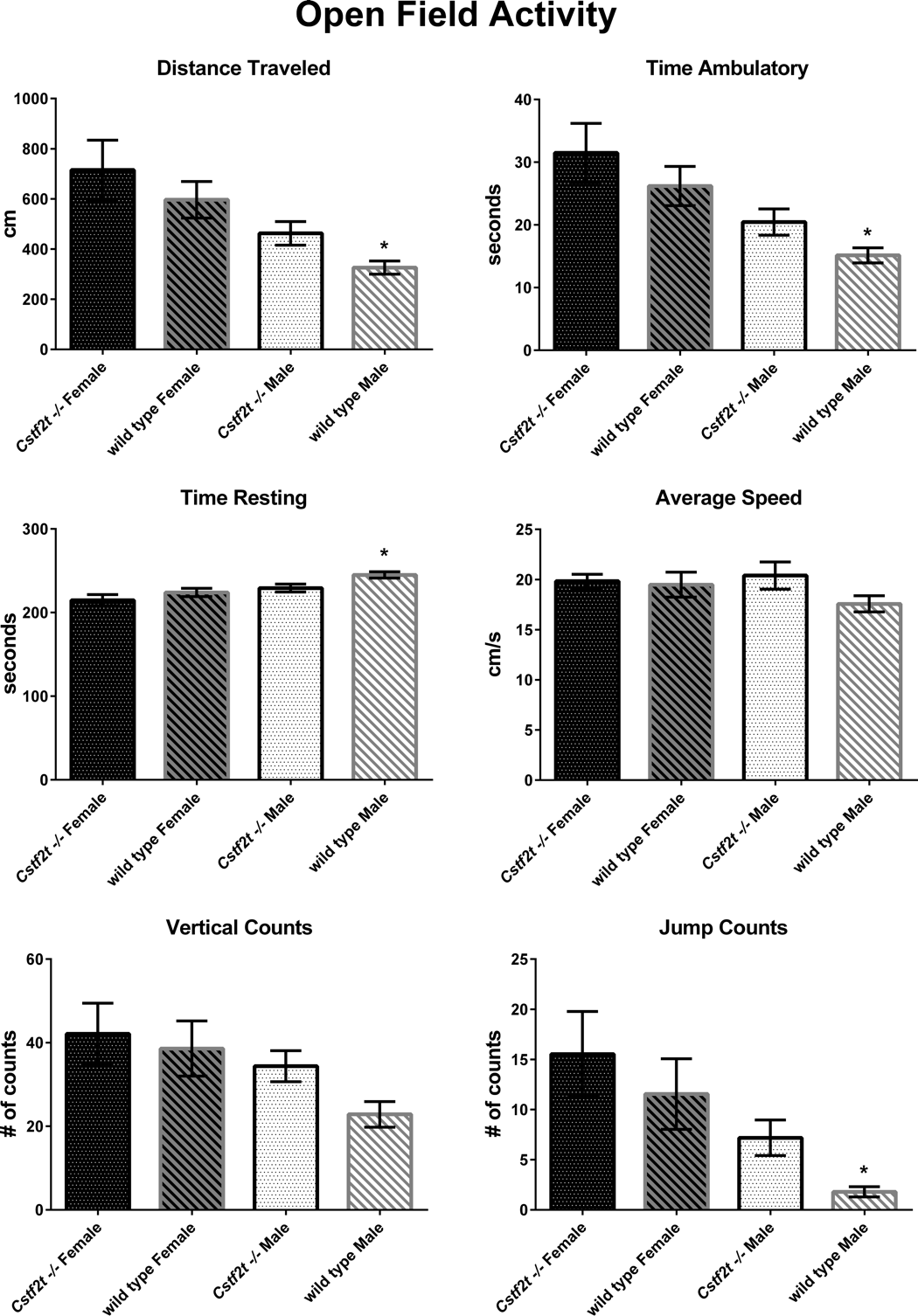

Supplement: S2 Fig — Values represent mean ± SEM. 2-way ANOVA; Tukey’s post-hoc tests were completed. *p<0.05, Cstf2t-/- vs. wild type mice. Parameters were Distance Traveled, Time Ambulatory, Time Resting, Average Speed, Vertical Counts, and Jump Counts. (TIF) [file pone.0165976.s002.tif]

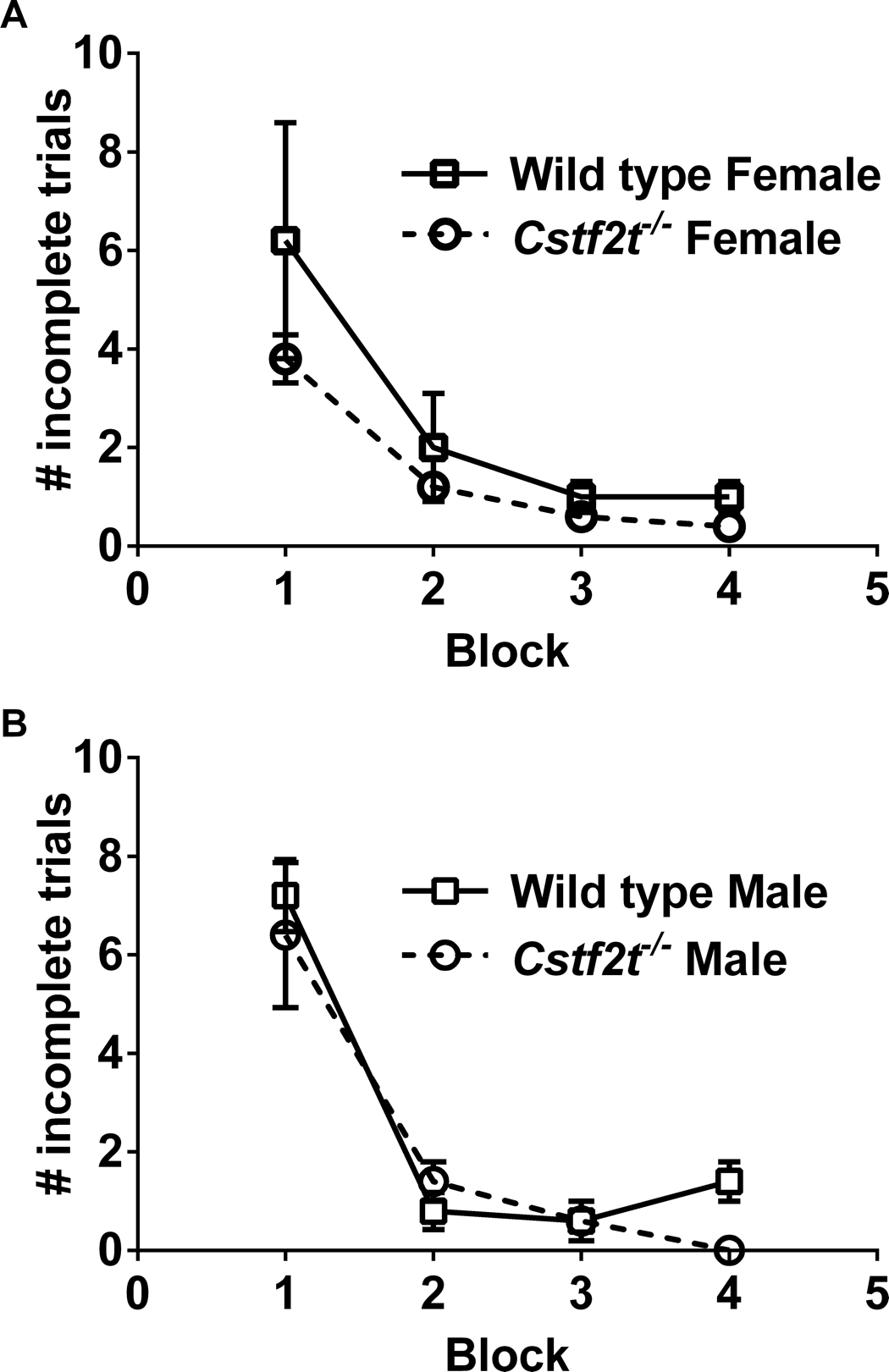

Supplement: S3 Fig — (A) Comparison of female groups using the “number of incomplete trials” metric, an estimate of maze task participation. There were no significant differences between groups. (B) Comparison of male groups examining “number of incomplete trials.” There were no significant differences between groups. (TIF) [file pone.0165976.s003.tif]

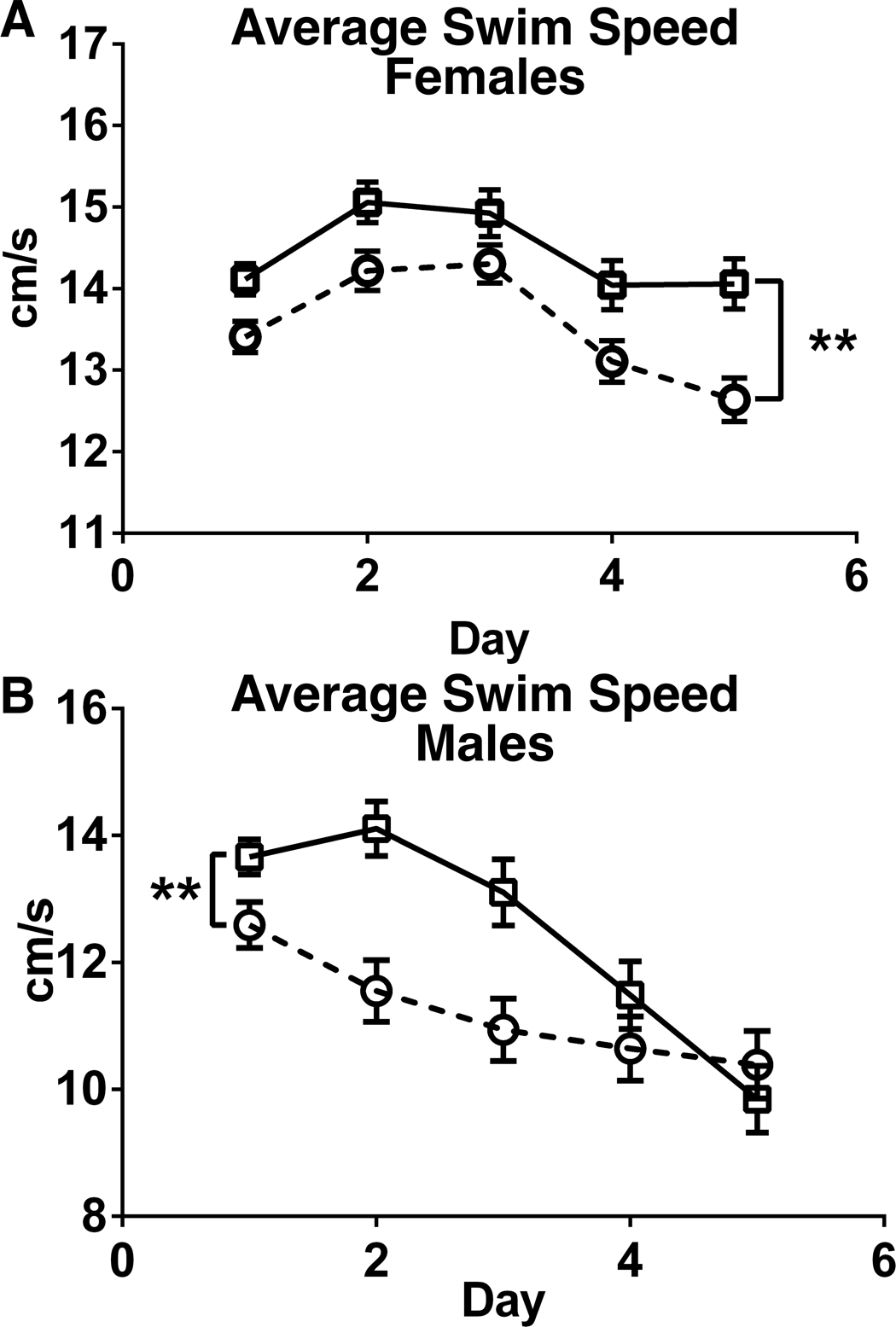

Supplement: S4 Fig — Female and male Cstf2t-/- and wild type mice (185 ± 10 days of age) were trained in a MWM four times per day for 5 days followed by reference memory trials 24 hours post training. (A) Examination of differences in average group swim speed between female groups showed a significant difference between groups (2-way ANOVA (F (3, 1320) = 35.00; p < 0.0001)). A post-hoc Tukey’s multiple comparison test revealed a significant difference between Cstf2t-/- and wild type mice (p < 0.0001). (B) Examination of differences in average group swim speed between male groups showed a significant difference between groups (2-way ANOVA (F (3, 1240) = 19.30; p < 0.0001)). A post-hoc Tukey’s multiple comparison test revealed significant differences between wild type mice and Cstf2t-/- mice (p < 0.0001). All data are presented as mean ± SEM. Data shown are for 185 groups only. ANOVA analyses were performed on all groups. (TIF) [file pone.0165976.s004.tif]
